# dmf‐g16: A Gaussian Wrapper for Reliable Double‐Ended Transition‐State Searches With Native Input Formats

**DOI:** 10.1002/jcc.70378

**Published:** 2026-05-04

**Authors:** Shin‐ichi Koda, Shinji Saito

**Affiliations:** ^1^ Department of Theoretical and Computational Molecular Science Institute for Molecular Science, National Institutes of Natural Sciences Okazaki Japan; ^2^ Molecular Science Program Graduate University for Advanced Studies, SOKENDAI Okazaki Japan

**Keywords:** direct MaxFlux method, flat‐bottom elastic network model, Gaussian, PyDMF, transition‐state searches

## Abstract

Transition‐state (TS) searches are central to computational studies of chemical reactions, yet advanced methods often require substantial effort to integrate into routine workflows. Consequently, users tend to rely on familiar software and established input formats. Here, we present dmf‐g16, a Gaussian‐specific front end to the Direct MaxFlux (DMF) reaction‐path optimization method implemented in PyDMF. dmf‐g16 enables DMF‐based TS searches with minimal workflow changes: users simply replace the Gaussian executable with dmf‐g16, while native QST2/QST3 input files remain unchanged. For QST inputs, DMF performs explicit path optimization using Gaussian as an external energy calculator, followed by TS refinement in Gaussian from the highest‐energy path point. Benchmarks on 121 reactions show a substantial improvement in reliability over Gaussian QST2, increasing the success rate from 31.4% to 93.4%. Although path optimization adds computational cost, wall‐clock time is typically only a few times that of QST2 and can be reduced through parallel energy evaluation.

## Graphical abstract

Text: dmf‐g16 makes transition state searches with Gaussian easier and more reliable by allowing users to simply replace the Gaussian executable while keeping existing QST2 or QST3 input files unchanged, with Gaussian used as an external energy and gradient engine. Benchmarks on 121 reactions demonstrate a substantial improvement in reliability over Gaussian QST2, increasing the success rate from 31.4% to 93.4%.

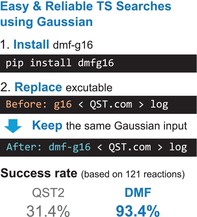

## 1 Introduction

Most molecular transformations can be described as processes that cross energy barriers on a potential energy surface. Therefore, identifying the transition state (TS), that is, a first‐order saddle point, is essential for understanding their mechanisms. However, TSs are inherently short‐lived and are difficult to observe directly in experiments. As a result, computational methods are widely used to search for TSs [1].

Computational methods for TS searches are broadly divided into single‐ended and double‐ended methods. Single‐ended methods start from an initial TS guess and optimize a single structure [1]. In contrast, double‐ended methods construct a reaction path connecting the reactant and product structures and search for TSs along this path [1, 2, 3]. A major advantage of double‐ended methods is that only the stable structures at both ends are required as input, thus no prior TS guess is needed. However, optimizing the full reaction path is often computationally expensive. In practice, the two approaches are complementary, and a TS‐like structure obtained by a double‐ended method is commonly refined using a single‐ended method [4, 5].

Owing to their importance, TS search methods continue to be actively developed. We have recently developed the Direct MaxFlux method (DMF) [6], a variational double‐ended reaction‐path optimization approach that enables efficient path optimization using a relatively small number of energy evaluations by directly minimizing the original MaxFlux objective function [7, 8]. In addition, by combining DMF with a structure‐based potential energy, the flat‐bottom elastic network model (FB‐ENM) [9, 10], we developed a method that rapidly and reliably generates chemically plausible initial paths for subsequent accurate TS searches. The Python implementation of these methods, PyDMF [11], provides a general interface based on the Atomic Simulation Environment (ASE) [12] and is designed to use a wide range of materials science software packages as engines for energy evaluations.

Although many advanced computational methods have been developed, there is often a gap between their development and their widespread adoption by users. In practical computation, users rely on software packages together with their customized input formats, scripts, and workflows. Consequently, beyond theoretical performance and computational efficiency, compatibility with existing environments and low adoption cost are critical factors in the acceptance of new methods. Methods that require substantial changes to familiar inputs or workflows, therefore, face significant barriers to widespread use.

For example, Gaussian [13], a quantum chemistry package widely used by both experimental and theoretical researchers, offers a double‐ended transition‐state (TS) search method based on the Quadratic Synchronous Transit (QST) approach, as implemented in Gaussian in the 1990s [14, 15]. Despite several decades of continued development of alternative TS search methods [4, 5, 6, 7, 8, 9, 10, 16, 17, 18, 19, 20, 21, 22, 23, 24, 25, 26, 27, 28, 29, 30, 31, 32, 33], QST remains the standard double‐ended TS search option within Gaussian and continues to be widely used in practice, reflecting the strong reliance of users on established Gaussian‐based workflows and the substantial cost associated with transitioning to methods that are not natively supported.

dmf‐g16 introduced in this paper is a Gaussian‐specific front end for DMF. It performs TS searches using DMF, while running Gaussian externally to obtain energies and gradients. The design goal of dmf‐g16 is twofold: to minimize adoption cost and to preserve existing workflows. Minimal adoption cost is achieved by requiring only installation and a slight modification of existing calculation scripts, namely replacing the Gaussian executable name (e.g., g16) with dmf‐g16. At the same time, existing workflows are preserved because Gaussian native input files can be used without modification.

To perform calculations, users simply provide dmf‐g16 with Gaussian native input files in the conventional QST2/QST3 format. dmf‐g16 first parses the endpoint structures (and the user‐specified intermediate point in a QST3 input) together with the computational settings. It then performs reaction‐path optimization using PyDMF with Gaussian as the energy evaluation engine. Finally, dmf‐g16 carries out TS optimization in Gaussian using the highest‐energy structure along the optimized path as the initial guess. In the latter part of this paper, we compare the performance of the Gaussian native QST method and dmf‐g16 in TS searches using identical input files.

## 2 User Overview

This section outlines how end users interact with dmf‐g16, which performs TS searches based on DMF by running Gaussian externally. Detailed usage instructions are available in the Supporting Information and the dmf‐g16 GitHub repository [34].

To minimize the adoption cost, dmf‐g16 is designed around the following principles:

- Easy installation
- Minimal modification of Gaussian execution scripts
- Use of Gaussian native input files

Each principle is briefly outlined below.

### 2.1 Installation

dmf‐g16 is written in Python and can be easily installed in a standard Python environment using conda. Listing 1 presents a minimal installation script that creates a conda virtual environment, which is recommended for convenient installation of the dependency cyipopt [35], the Python wrapper of the general‐purpose optimizer IPOPT [36] used in reaction‐path optimization. Both dmf‐g16 and its DMF backend, PyDMF, are distributed via PyPI and can be installed using pip.

LISTING 1 **A minimal installation script for dmf‐g16 using a conda virtual environment, followed by installation of dmf‐g16 via pip.**

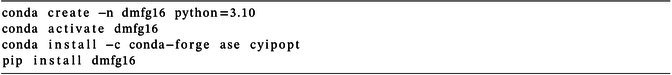

### 2.2 Modification of the Gaussian Execution Scripts

Running dmf‐g16 requires only a minimal modification of an existing Gaussian execution script. First, the conda virtual environment in which dmf‐g16 is installed (here named dmfg16) is activated, either before executing the script or at the beginning of the script:

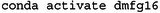

Next, the Gaussian executable specified in the script is replaced with dmf‐g16. For example, a standard command

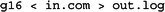

can be replaced by

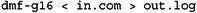

If the Gaussian executable has a different name, it can be specified explicitly using the ––exe option, for example,

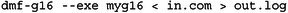

The default command name and settings of dmf‐g16 are chosen for convenience to match the current Gaussian version, but other versions are also supported. For example, Gaussian 09 can be used as

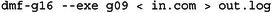

### 2.3 Use of Gaussian Native Input Files

As implied by the script modification described above, dmf‐g16 uses Gaussian native input files without modification. This allows users to directly reuse inputs created with tools such as GaussView [37], thereby preserving existing workflows.

When a Gaussian input file in the QST2/QST3 format is provided, dmf‐g16 does not run the original QST calculation. Instead, it performs a two‐step procedure:

- reaction‐path optimization based on DMF
- TS optimization in Gaussian using the highest‐energy structure along the optimized path as the initial structure

If the provided input file is not in the QST format, dmf‐g16 simply passes it to Gaussian without modification, so arbitrary Gaussian jobs can also be executed through dmf‐g16. The standard output of dmf‐g16 corresponds to that of the Gaussian job ultimately executed. When a QST‐formatted input is used, log files from the first step are saved in a separate working directory (see Supporting Information for details).

In the second step for a QST‐formatted input, the Gaussian input file is generated by modifying the original input in only two places. In the route section, the qst2 or qst3 option in the opt keyword is replaced with ts. The molecular coordinates, which appear multiple times in a QST input, are replaced with a single set corresponding to the highest‐energy point along the optimized path. Listing 2 shows an example of the original and modified inputs; apart from the color‐highlighted sections, the input file remains unchanged. It may therefore be helpful to specify calculation settings with the understanding that the transformed job will ultimately be executed.

LISTING 2 **Example of a Gaussian QST2‐formatted input file and the corresponding input file generated and executed by dmf‐g16. Only the highlighted sections are modified during the conversion.**

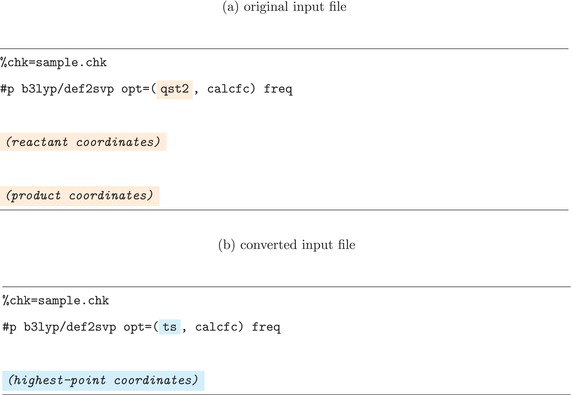

## 3 Implementation

**FIGURE 1:**
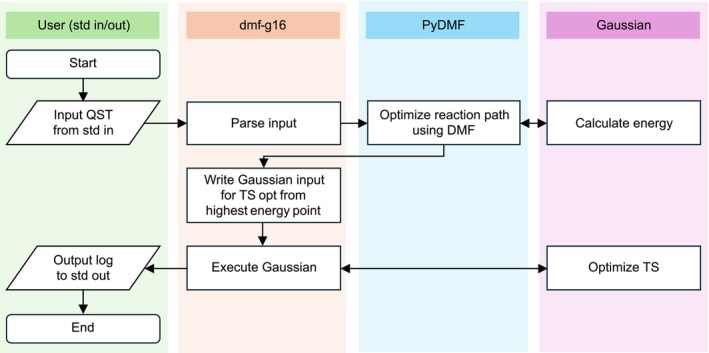
Flowchart of dmf‐g16 for DMF‐based TS searches. dmf‐g16 receives a Gaussian QST‐formatted input from standard input, parses the structures and computational settings, and passes them to PyDMF for DMF‐based reaction‐path optimization in cooperation with Gaussian for energy evaluations. After path optimization, dmf‐g16 generates a Gaussian input for TS optimization from the highest‐energy point along the path, executes Gaussian externally, and forwards the resulting output to standard output.

This section outlines the design of dmf‐g16. Figure 1 illustrates the workflow. dmf‐g16 operates in coordination with PyDMF [11], which serves as a general backend for DMF calculations, and with Gaussian [13], which performs energy evaluations. dmf‐g16, PyDMF, and Gaussian function as three independent applications that interact with one another. PyDMF is designed to be general and abstract and is based on the Atomic Simulation Environment (ASE) [12]. While this design enables broad compatibility, it also entails a relatively high initial learning cost for end users. dmf‐g16 acts as a wrapper that allows Gaussian users to access PyDMF without directly interacting with PyDMF or ASE.

When a Gaussian input in the QST2/QST3 format is provided to dmf‐g16 via standard input, dmf‐g16 parses the input to extract the reactant and product coordinates (and, for QST3, the user‐specified intermediate point), together with computational settings such as the electronic structure method and basis set. These data are then passed to PyDMF. Because the Gaussian parser in ASE does not support QST‐formatted inputs, a modified parser is used. Basic DMF parameters can also be specified through command‐line options of dmf‐g16 (see the Supporting Information for details).

PyDMF first generates an initial reaction path using FB‐ENM [9, 10] and then performs reaction‐path optimization based on DMF in cooperation with Gaussian. In this process, Gaussian is used solely for energy evaluations, while PyDMF handles output parsing, path updates, and the external execution of Gaussian for subsequent evaluations.

After the reaction‐path optimization is completed, dmf‐g16 generates a Gaussian input file for TS optimization by modifying only a few parts of the original QST‐formatted input, as described in the previous section. The highest‐energy structure along the optimized path is used as the initial structure. Gaussian is then executed externally using the subprocess module, and its standard output is forwarded as the standard output of dmf‐g16.

In summary, dmf‐g16 internalizes PyDMF while preserving existing Gaussian workflows. By parsing Gaussian native QST‐formatted inputs and handling input and output through standard streams in the same manner as Gaussian, dmf‐g16 enables DMF‐based TS searches with minimal changes to existing execution scripts.

## 4 Results and Discussion

We next perform TS searches using Gaussian native QST and the DMF‐based approach implemented in dmf‐g16, starting from the same input files. Rather than comparing the underlying algorithms themselves, this study focuses on the practical benefit of dmf‐g16, specifically how much TS search performance can be improved by changing only the executable name, while leaving the input files and overall workflow unchanged. In the following subsections, we examine TS searches based on QST2 inputs, which specify only reactant and product structures, as well as QST3 inputs, which additionally include a user‐specified intermediate point.

### 4.1 QST2

TS searches were performed for 121 reactions curated through several studies [4, 5, 38], containing 3 to 52 main‐group atoms. All calculations were carried out using DFT with the B3LYP‐D3BJ functional [39, 40, 41, 42, 43] and the def2‐SVP basis set [44, 45]. For both Gaussian native QST2 calculations and the TS optimizations performed in dmf‐g16, the following Gaussian optimization options were applied in addition to qst2: calcfc, which computes the analytical Hessian only once at the beginning; noeigentest, which disables eigenvalue consistency checks during the optimization; and maxstep = 15, which reduces the maximum step size to half of the default value. The corresponding input files are provided in Supporting Information. After TS optimization, the analytical Hessian was recomputed, and a search was counted as successful if exactly one imaginary frequency was obtained.

**FIGURE 2:**
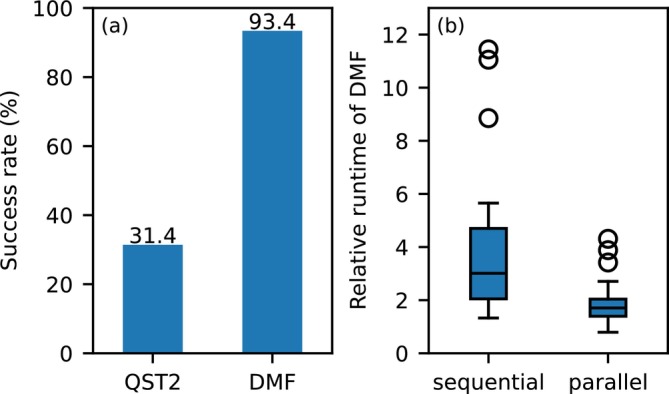
Performance of TS searches using Gaussian native QST2 and dmf‐g16. (a) Success rates of TS searches for 121 reactions using Gaussian native QST2 and the DMF‐based approach implemented in dmf‐g16, (b) Relative wall‐clock times for the 38 reactions successfully treated by both methods, reported relative to QST2. Sequential DMF calculations using 32 CPU cores and parallel DMF calculations using 64 CPU cores with the ––parallel option are compared.

Figure 2a shows the success rates of TS searches. Using Gaussian native QST2, TSs were successfully located for 38 reactions (31.4%). In contrast, the DMF‐based approach implemented in dmf‐g16 achieved successful TS searches for 113 reactions (93.4%). Several unsuccessful cases were nearly converged but exhibited small oscillations. By adding the gdiis option and repeating the optimizations, the number of successful TS searches with dmf‐g16 further increased to 116 reactions (95.9%). Note that the gdiis option is not available for Gaussian native QST jobs. These results demonstrate a substantial improvement in the reliability of TS searches when using dmf‐g16.

Wall‐clock times were compared for the 38 reactions for which both QST2 and DMF‐based TS searches were successful (Figure 2b). To account for the strong dependence of absolute timings on molecular size, wall times are reported relative to QST2, which was set to unity.

When calculations were performed sequentially using 32 CPU cores, DMF typically required about two to four times longer wall time than QST2, aside from a few outliers. This difference is expected because QST2 does not perform reaction‐path optimization, whereas DMF explicitly optimizes a reaction path by evaluating multiple structures along the path.

Importantly, DMF is known to be efficient among reaction‐path optimization methods [6], requiring far fewer energy evaluations than widely used approaches such as the nudged elastic band (NEB) method [5, 16, 17, 18, 19, 46]. In other words, despite performing reaction‐path optimization, a wall time of only two to four times that of QST2 is remarkably short.

dmf‐g16 supports parallel evaluation of energies at multiple points along the reaction path using the ‐p or ––parallel option. When a sufficient number of CPU cores is available such that the parallel efficiency of single‐point energy calculations becomes saturated, distributing computational resources over multiple path points improves overall efficiency. For example, with 64 CPU cores, DMF calculations typically required less than twice the wall time of QST2 calculations using the same number of cores.

### 4.2 QST3

**FIGURE 3:**
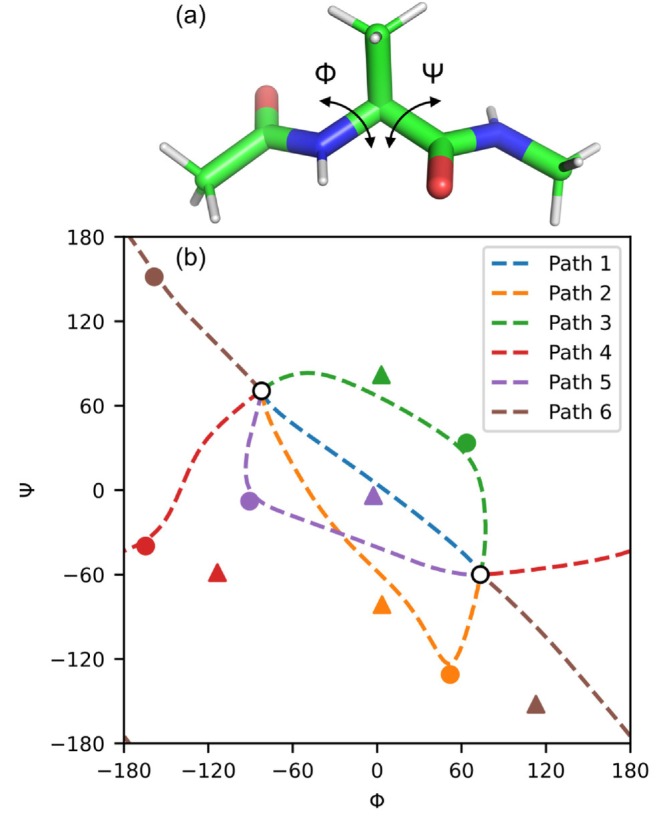
QST3‐like TS searches for conformational transitions of alanine dipeptide using dmf‐g16. (a) Molecular structure of alanine dipeptide illustrating the backbone dihedral angles Φ and Ψ. (b) Reaction paths projected onto the (Φ,Ψ) dihedral‐angle space: white circles denote the two stable endpoint conformations; colored circles indicate user‐specified intermediate points used in QST3‐formatted inputs; dashed lines represent the initial paths generated by FB‐ENM interpolation in PyDMF; triangles mark the TS structures obtained after DMF‐based path optimization and subsequent TS refinement in Gaussian. Path 1 corresponds to a QST2‐formatted input without an intermediate point.

dmf‐g16 supports TS searches using Gaussian native QST3‐formatted inputs, which specify both endpoints of a reaction path together with a user‐defined intermediate point. This feature is particularly useful when approximate TS geometries are known in advance or when multiple reaction paths are plausible. Here, we demonstrate QST3‐like TS searches using conformational transitions of alanine dipeptide (Figure 3a).

Alanine dipeptide exhibits multiple conformers associated with the dihedral angles of its two peptide bonds, giving rise to diverse transition paths. Two stable conformations were selected as the endpoints (white circles in Figure 3b), and several QST3‐formatted inputs with different intermediate points (colored circles) were prepared (see Supporting Information). The dashed lines in Figure 3b show the initial paths generated by FB‐ENM interpolation in PyDMF. For comparison, Path 1 was generated from a QST2‐formatted input without an intermediate point.

Subsequent DMF‐based path optimization, followed by TS refinement in Gaussian, successfully yielded five distinct TS structures without failure. Although Path 1 and Path 5 converged to the same TS, all TSs were located close to their corresponding initial paths. These results demonstrate that dmf‐g16 can reliably perform QST3‐like TS searches and identify TSs near paths that pass through user‐specified intermediate points.

For comparison, Gaussian native QST3 calculations using the same input files succeeded in locating TSs in four of six cases, indicating that QST3 can successfully locate TSs when high‐quality intermediate structures are provided. Nevertheless, the present results do not indicate reliability comparable to that observed for dmf‐g16.

In practice, the Gaussian native QST3 method has often been used to increase the success rate of TS searches when QST2 fails. In contrast, dmf‐g16 exhibits consistently high success rates for both QST2‐like and QST3‐like TS searches. Under these circumstances, QST3‐like searches are no longer limited to trial‐and‐error strategies for locating a single TS, but instead gain increased value as a systematic tool for exploring and comparing multiple competing reaction mechanisms, as demonstrated in the present example.

## 5 Conclusions

In this work, we presented dmf‐g16, a Gaussian‐specific front end for the Direct MaxFlux method (DMF) [6, 9, 10], designed to enable reliable TS searches with minimal changes to existing Gaussian workflows. By requiring only a slight modification of execution scripts and allowing Gaussian native QST‐formatted input files to be used without alteration, dmf‐g16 significantly lowers the adoption barrier for DMF‐based TS searches while preserving familiar user practices.

More generally, this work highlights the importance of combining a specialized, user‐friendly front end with a general and extensible computational backend. By encapsulating the abstract and flexible design of PyDMF [11] behind a Gaussian‐specific interface, dmf‐g16 enables advanced reaction‐path optimization methods to be used in routine practice without exposing end users to unnecessary complexity. Similar design principles also apply to alternative front‐end implementations built on top of PyDMF. One example is ColabReaction [47], a web‐based interface that couples PyDMF with external machine‐learning potentials [48, 49]. Such applications further demonstrate the versatility of combining a general reaction‐path backend with specialized user interfaces.

Benchmark calculations using QST2 inputs demonstrate that dmf‐g16 substantially improves the reliability of TS searches compared to the Gaussian native QST method [15]. As expected, this improvement comes at the cost of increased computational time, since DMF explicitly performs reaction‐path optimization, whereas QST does not. However, the resulting wall‐clock time is typically only a few times longer than that of QST, which is remarkably short for a reaction‐path optimization method.

From a practical perspective, the increase in computational cost is often justified by the improved reliability, which substantially reduces the need for repeated calculations and manual intervention. In many practical applications, QST calculations require repeated remodeling of input structures and multiple trial calculations before a satisfactory TS is obtained. When such iterative user intervention is taken into account, the overall burden in terms of both computational time and manual modeling effort can easily exceed that of a single, more robust DMF‐based TS search.

Moreover, when sufficient computational resources are available, the wall‐time difference between QST and DMF‐based TS searches can be further reduced by parallelizing energy evaluations along the reaction path using the ––parallel option in dmf‐g16. Under such conditions, DMF‐based TS searches can approach the wall time of QST while retaining their higher reliability, making them particularly attractive in modern high‐performance computing environments.

Overall, dmf‐g16 provides a practical and reliable alternative to Gaussian native QST for TS searches. By combining improved robustness with minimal workflow disruption and efficient use of computational resources, it offers a realistic means of integrating advanced reaction‐path optimization methods into routine computational chemistry practice.

## Supporting information




**Data S1.** Supporting Information S1.

**Data S2.** Supporting Information S2.

## Data Availability

dmf‐g16 is freely available at the Python Package Index (PyPI) under the package name dmfg16, and the source code is available on GitHub at https://github.com/shin1koda/dmf‐g16. All results reported in this study can be reproduced using dmf‐g16 and the QST2/QST3‐formatted Gaussian input files provided as Supporting Information.
