# Supplementary material for: dmf‐g16: A Gaussian Wrapper for Reliable Double‐Ended Transition‐State Searches With Native Input Formats
Source: J Comput Chem. 2026 May 4;47:e70378. doi: 10.1002/jcc.70378 (PMC13138099; doi:10.1002/jcc.70378)
Supplement: Supplementary file 1 — Data S1. Supporting Information S1. [file JCC-47-0-s001.pdf]

Supporting Information for "dmf-g16: A Gaussian Wrapper for Reliable Double-Ended Transition-State Searches with Native Input Formats"

Shin-ichi Koda*,†,‡ and Shinji Saito*,†,‡

†*Department of Theoretical and Computational Molecular Science, Institute for Molecular Science, Myodaiji, Okazaki, Aichi 444-8585, Japan*

‡*School of Physical Sciences, The Graduate University for Advanced Studies, Myodaiji, Okazaki, Aichi 444-8585, Japan*

E-mail: koda@ims.ac.jp; shinji@ims.ac.jp

Contents

S1 Usage of `dmf-g16` **S2**

S1.1 Command-line options S2

S1.2 Example usage S4

S2 Files in the working directory **S5**

S3 Limitations **S6**

References **S6**

S1 Usage of `dmf-g16`

This section summarizes the basic usage of `dmf-g16`, a Gaussian[1]-specific front end for the Direct MaxFlux (DMF) reaction-path optimization method[2–4] implemented in `PyDMF`.[5] Installation procedures and modification of calculation scripts are described in the main text.

S1.1 Command-line options

The command-line options listed below control the behavior of `dmf-g16` through a subset of functionalities provided by `PyDMF`. These options are intended to cover common and essential use cases. For more advanced control of the direct MaxFlux method (DMF), users are encouraged to write Python scripts directly using the `PyDMF` API, rather than relying on the `dmf-g16` command-line interface.

`-n`, `--npoints`

Number of energy evaluation points sampled between the two endpoints of the reaction path. The endpoint structures themselves are excluded from this count. Energies are evaluated at these intermediate points along the reaction path, and the resulting energy profile is used to identify the highest-energy structure, which serves as the initial transition-state (TS) guess. In general, increasing the number of evaluation points improves the TS-guess structure, whereas the computational cost scales approximately linearly with the number of energy evaluation points. The default value is `3`.

`-e`, `--equidistant`

If this flag is specified, the distance between adjacent energy evaluation points along the reaction path is kept equal. Without this option, energy evaluation points are preferentially distributed toward higher-energy regions of the reaction path, providing increased resolution around the transition-state region and enabling a more reliable TS search. This option is particularly useful when combined with a relatively large

number of evaluation points, as it allows for a more uniform overview of the entire reaction path.

`-t, --tol`

Convergence criterion for stopping the Direct MaxFlux (DMF) optimization. Three predefined levels are available: `loose`, `middle`, and `tight`. The default setting is `middle`, which balances computational cost and the robustness of the generated TS guess. For reactions involving relatively simple structural changes, the `loose` setting is often sufficient, whereas the `tight` setting is recommended for more complex reactions. See the original paper[2] for details.

`-d, --dir`

Working directory used for DMF calculations. All intermediate files and results generated during the DMF optimization are stored in this directory. The default directory name is `dmf`. When running multiple calculations concurrently or sequentially, users should ensure that the working directory names do not conflict to avoid unintended overwriting of files.

`--exe`

Name of the Gaussian executable used to perform quantum chemical calculations. This option allows the user to specify a non-default Gaussian command. The default value is `g16`.

`-P, --parallel`

Enable parallel execution at the DMF level. When this option is specified, calculations at all energy evaluation points are distributed across the available computational resources and executed concurrently. This option is particularly recommended when sufficient computational resources are available such that the parallel efficiency of individual single-point calculations is already saturated. The computational resources must be explicitly specified either via `nprocshared` or `cpu` in the Gaussian Link0 section,

or through the `cdef` environment variable. At present, parallel execution is supported only within a single CPU node; inter-node parallelization using Linda workers and GPU acceleration are not supported.

`-C, --only-ts-guess`

Terminate the workflow after the TS-guess generation. When this option is specified, the input file for the subsequent Gaussian `opt=ts` optimization is generated, but the optimization itself is not executed.

`-R, --restart`

Restart a DMF optimization using the reaction path data remaining in the working directory. When this option is specified, the stored reaction path is reused as the initial path for a new DMF optimization, rather than continuing strictly from the last completed DMF iteration. Specifically, if the path expansion coefficients file (`coefs.npy`) is available, the reaction path is reconstructed from these coefficients; otherwise, if an intermediate path file such as `imagesN.log` is present, a reaction path passing through the stored structures is constructed; if neither file is found, the default initial reaction path is generated, which is equivalent to running the calculation without the `--restart` option. When this option is specified, any intermediate structures provided in a QST3-formatted input are ignored.

S1.2 Example usage

```
dmf-g16 < qst.com > logfile
```

Standard usage of `dmf-g16`, where a QST-formatted input is read from standard input and the output is written to a log file.

```
dmf-g16 --exe myg16 < qst.com > logfile
```

Use a non-default Gaussian executable named `myg16`.

```
dmf-g16 -n 5 -P --tol loose --dir work < qst.com > logfile
```

Perform TS-guess generation using five energy evaluation points with DMF-level parallel execution and a loose convergence criterion. All intermediate files are stored in the working directory `work`.

```
dmf-g16 -n 10 --equidistant -C < qst.com > logfile
```

Sample ten energy evaluation points uniformly along the reaction path. The Gaussian `opt=ts` optimization is skipped, and only the TS-guess input file is generated.

```
dmf-g16 --restart < qst.com > logfile
```

Restart a DMF optimization using reaction path data remaining in the working directory.

S2 Files in the working directory

This section describes files generated in the working directory during DMF reaction path optimization. These files store intermediate data from the optimization and can be reused for restarting or analyzing DMF calculations.

`images`*N*`.com, .log, .chk`

Gaussian input, output, and checkpoint files used to evaluate the N-th energy evaluation point.

`ipopt_fbenm`*I*`.log`

Log file recording the progress of the FB-ENM interpolation between the I-th and (I+1)-th reference structures. See the IPOPT[6] documentation for details.

`ipopt_dmf.log`

Log file recording the progress of the DMF path optimization. See the IPOPT[6] documentation for details.

`coefs.npy`

Binary NumPy file containing the final expansion coefficients of the reaction path representation used in DMF optimization.

`dmf.traj`

Atomic Simulation Environment (ASE)[7] trajectory file that contains the final energy evaluation points.

S3 Limitations

Gaussian is a highly versatile software package with a broad range of functionalities developed over many years. Consequently, `dmf-g16` cannot fully support all features available in Gaussian. Since `dmf-g16` and `PyDMF` are based on ASE,[7] Gaussian features not supported by ASE are generally unavailable. For example, ONIOM calculations are currently not supported.

References

(1) Frisch, M. J.; Trucks, G. W.; Schlegel, H. B.; Scuseria, G. E.; Robb, M. A.; Cheeseman, J. R.; Scalmani, G.; Barone, V.; Petersson, G. A.; Nakatsuji, H.; Li, X.; Caricato, M.; Marenich, A. V.; Bloino, J.; Janesko, B. G.; Gomperts, R.; Mennucci, B.; Hratchian, H. P.; Ortiz, J. V.; Izmaylov, A. F.; Sonnenberg, J. L.; Williams-Young, D.; Ding, F.; Lipparini, F.; Egidi, F.; Goings, J.; Peng, B.; Petrone, A.; Henderson, T.; Ranasinghe, D.; Zakrzewski, V. G.; Gao, J.; Rega, N.; Zheng, G.; Liang, W.; Hada, M.; Ehara, M.; Toyota, K.; Fukuda, R.; Hasegawa, J.; Ishida, M.; Nakajima, T.; Honda, Y.; Kitao, O.; Nakai, H.; Vreven, T.; Throssell, K.; Montgomery, J. A., Jr.; Peralta, J. E.; Ogliaro, F.; Bearpark, M. J.; Heyd, J. J.; Brothers, E. N.; Kudin, K. N.; Staroverov, V. N.; Keith, T. A.; Kobayashi, R.; Normand, J.; Raghavachari, K.; Ren-

dell, A. P.; Burant, J. C.; Iyengar, S. S.; Tomasi, J.; Cossi, M.; Millam, J. M.; Klene, M.; Adamo, C.; Cammi, R.; Ochterski, J. W.; Martin, R. L.; Morokuma, K.; Farkas, O.; Foresman, J. B.; Fox, D. J. Gaussian 16 Revision C.01. 2016; Gaussian Inc. Wallingford CT.

(2) Koda, S.-i.; Saito, S. Locating Transition States by Variational Reaction Path Optimization with an Energy-Derivative-Free Objective Function. *Journal of Chemical Theory and Computation* **2024**, *20*, 2798–2811.

(3) Koda, S.-i.; Saito, S. Flat-Bottom Elastic Network Model for Generating Improved Plausible Reaction Paths. *Journal of Chemical Theory and Computation* **2024**, *20*, 7176–7187.

(4) Koda, S.-i.; Saito, S. Correlated Flat-Bottom Elastic Network Model for Improved Bond Rearrangement in Reaction Paths. *Journal of Chemical Theory and Computation* **2025**, *21*, 3513–3522.

(5) Koda, S.-i. A Python implementation of the direct MaxFlux method. `https://github.com/shin1koda/dmf`, accessed: 2025-01-16.

(6) Wächter, A.; Biegler, L. T. On the implementation of an interior-point filter line-search algorithm for large-scale nonlinear programming. *Mathematical Programming* **2006**, *106*, 25–57.

(7) Larsen, A. H.; Mortensen, J. J.; Blomqvist, J.; Castelli, I. E.; Christensen, R.; Dułak, M.; Friis, J.; Groves, M. N.; Hammer, B.; Hargus, C.; Hermes, E. D.; Jennings, P. C.; Jensen, P. B.; Kermode, J.; Kitchin, J. R.; Kolsbjerg, E. L.; Kubal, J.; Kaasbjerg, K.; Lysgaard, S.; Maronsson, J. B.; Maxson, T.; Olsen, T.; Pastewka, L.; Peterson, A.; Rostgaard, C.; Schiøtz, J.; Schütt, O.; Strange, M.; Thygesen, K. S.; Vegge, T.; Vilhelmsen, L.; Walter, M.; Zeng, Z.; Jacobsen, K. W. The atomic simulation environment—a

Python library for working with atoms. *Journal of Physics: Condensed Matter* **2017**, *29*, 273002.
